# Multiple ETS Family Proteins Regulate *PF4* Gene Expression by Binding to the Same ETS Binding Site

**DOI:** 10.1371/journal.pone.0024837

**Published:** 2011-09-12

**Authors:** Yoshiaki Okada, Haruaki Nobori, Mikiko Shimizu, Miho Watanabe, Masaaki Yonekura, Tomoko Nakai, Yuko Kamikawa, Atsuko Wakimura, Nobuaki Funahashi, Hiroki Naruse, Ayako Watanabe, Daisuke Yamasaki, So-ichiro Fukada, Kazuta Yasui, Kayoko Matsumoto, Takahiro Sato, Kenji Kitajima, Toru Nakano, William C. Aird, Takefumi Doi

**Affiliations:** 1 Graduate School of Pharmaceutical Sciences, Osaka University, Osaka, Japan; 2 Graduate School of Frontier Biosciences, Osaka University, Osaka, Japan; 3 Department of Research, Osaka Red Cross Blood Center, Osaka, Japan; 4 Division of Cord Blood Cell Processing, Osaka Red Cross Blood Center, Osaka, Japan; 5 Department of Cellular Physiological Chemistry, Tokyo Medical and Dental University, Tokyo, Japan; 6 Department of Pathology, Medical School, Osaka University, Osaka, Japan; 7 Center for Vascular Biology Research and Division of Molecular and Vascular Medicine, Beth Israel Deaconess Medical Center, Boston, Massachusetts, United States of America; Université Paris-Diderot, France

## Abstract

In previous studies on the mechanism underlying megakaryocyte-specific gene expression, several ETS motifs were found in each megakaryocyte-specific gene promoter. Although these studies suggested that several ETS family proteins regulate megakaryocyte-specific gene expression, only a few ETS family proteins have been identified. Platelet factor 4 (*PF4*) is a megakaryocyte-specific gene and its promoter includes multiple ETS motifs. We had previously shown that ETS-1 binds to an ETS motif in the PF4 promoter. However, the functions of the other ETS motifs are still unclear. The goal of this study was to investigate a novel functional ETS motif in the PF4 promoter and identify proteins binding to the motif. In electrophoretic mobility shift assays and a chromatin immunoprecipitation assay, FLI-1, ELF-1, and GABP bound to the −51 ETS site. Expression of FLI-1, ELF-1, and GABP activated the PF4 promoter in HepG2 cells. Mutation of a −51 ETS site attenuated FLI-1-, ELF-1-, and GABP-mediated transactivation of the promoter. siRNA analysis demonstrated that FLI-1, ELF-1, and GABP regulate *PF4* gene expression in HEL cells. Among these three proteins, only FLI-1 synergistically activated the promoter with GATA-1. In addition, only FLI-1 expression was increased during megakaryocytic differentiation. Finally, the importance of the −51 ETS site for the activation of the PF4 promoter during physiological megakaryocytic differentiation was confirmed by a novel reporter gene assay using in vitro ES cell differentiation system. Together, these data suggest that FLI-1, ELF-1, and GABP regulate *PF4* gene expression through the −51 ETS site in megakaryocytes and implicate the differentiation stage-specific regulation of *PF4* gene expression by multiple ETS factors.

## Introduction

Megakaryocytic differentiation is accompanied by drastic morphological changes that are induced by endomitosis and proplatelet formation. To understand the molecular mechanism of megakaryocyte-specific gene regulation during this unique differentiation process, several megakaryocyte-specific gene promoters, including the promoters of platelet factor 4 (PF4), c-Mpl, Glycoprotein (GP) IIb, GPV, GPIX, GPVI, GPIb and platelet basic protein (PBP), have been studied (reviewed in [1]). Each of these promoters includes multiple GATA and ETS motifs.

GATA-1 is shown to bind to the GATA motifs in most megakaryocyte-specific gene promoters. GATA-1 is one of the zinc finger transcription factors; it recognizes the T/AGATAA/G motif and promotes megakaryocytic and erythroid development. GATA-1 binds to GATA motifs in the megakaryocytic gene promoters and activates the gene expression (reviewed in [1]). On the other hand, multiple ETS family transcription factors, such as FLI-1 and PU.1, are known to bind to ETS motifs in each megakaryocyte-specific gene promoter. The members of the ETS family of transcription factors share an evolutionarily conserved DNA-binding domain of 85 amino acids with a winged-helix-turn-helix configuration [2]. ETS factors bind to GGAA/T core sequences. FLI-1 is known to be a positive regulator of megakaryocyte-specific gene expression. FLI-1 regulates several megakaryocyte-specific genes, including GPIIb, GPVI, GPIX and c-Mpl. FLI-1 interacts with GATA-1 and enhances its binding to DNA [3]. Homozygous FLI-1 knockout is embryonic lethal in mice and shows severe dysmegakaryopoiesis. PU.1 is known to be a transcriptional activator of the *GPIIb* and *PBP* genes [4], [5]. However, PU.1 is also reported to interact with GATA-1 and inhibit GATA-1 function through several possible mechanisms [6], [7], [8], [9], [10], [11]. This indicates that PU.1 may function as a negative regulator of megakaryocyte-specific gene expression.

To investigate the mechanism underlying megakaryocyte-specific gene expression, we have been studying the regulatory mechanism of the *PF4* gene expression by using the rat PF4 promoter. We demonstrated that several transcription factors (e.g. GATA-1, ETS-1, MEIS1, PBX1/2, PREP1 and USF1/2) bind to the proximal promoter region and activate the *PF4* gene expression [12], [13], [14], [15]. ETS-1 has been shown to activate the PF4 promoter through the −73 ETS site. However, the functions of several other ETS motifs in the PF4 promoter are still unknown. In the present study, we identified the −51 ETS site as a novel functional site in the promoter. The importance of the −51 ETS site for the activation of *PF4* gene expression in physiological megakaryocytic differentiation was demonstrated by a novel ES cell differentiation system. Based on EMSA and coexpression assays, 3 ETS family proteins, FLI-1, ELF-1, and GABP were shown to bind to the −51 ETS site and regulate the PF4 promoter. Analysis of expression patterns of *FLI-1*, *ELF-1*, and *GABP* during megakaryocytic differentiation suggested the differentiation stage-specific function of ETS factors. Thus, we succeeded in identifying multiple ETS family proteins that regulate megakaryocyte-specific *PF4* gene expression through the novel ETS site.

## Materials and Methods

### Preparation of plasmids and targeting vectors

The Rat PF4 promoter (PF4-luc) and human PF4 (hPF4-luc) reporter constructs were previously described [12], [14]. To generate the PF4 promoter deletion constructs (Del-500, Del-300, and Del-100), rat PF4 promoter upstream fragments were amplified by polymerase chain reaction (PCR) using 3 different primer sets (DNA sequences are shown in **Table S1**). The resulting fragments were phosphorylated by T4 DNA kinase and cloned into the *Sma*I site of PGV-B (TOYO B-Net, Tokyo, Japan). All DNA sequences were verified by automated DNA sequencing.

To generate the two PF4 promoter mutants containing a single mutation in −73 or −51 ETS site (−73 ETSmut and −51 ETSmut), an intermediate plasmid containing mutations in both sites (−73/−51 ETSmut) was prepared by two-step PCR amplification. In the first step, the rat PF4 promoter fragment was amplified by PCR using PF4-luc as a template and primers for the first PCR. In the second step, PCR was performed using primers (the first PCR product and a new primer) and PF4-luc as a template. The resulting 0.2-kb promoter fragment containing the −73 and −51 ETS mutations was digested with *Kpn*I and ligated with the 6.4-kb fragment of *Pvu*II-*Kpn*I digested PF4-luc. To generate the −51 ETSmut, −73/−51 ETSmut was digested with *Sma*I and the 0.7-kb fragment containing the −51 ETS mutation was ligated with the 5.9-kb fragment of *Sma*I-digested PF4-luc. To generate the −73 ETSmut, PF4-luc was digested with *Sma*I and the 0.7-kb fragment was ligated with the 5.9-kb fragment of *Sma*I-digested −73/−51 ETSmut. The DNA sequences of all the constructs were verified by automated DNA sequencing. All the primer sequences are shown in **Table S1**.

To generate FLI-1, PU.1, and GATA-1 expression vectors, cDNAs for FLI-1, PU.1, and GATA-1 were PCR amplified using HEL cDNA and gene-specific primers (shown in **Table S1**). The resulting PCR fragments were phosphorylated with T4 DNA kinase and cloned into the *Eco*RV site of pcDNA3 (Invitrogen, Carlsbad, CA). DNA sequences were verified by automated DNA sequencing. To generate the ELF-1 expression vector (pcDNA3-ELF-1), pCI-ELF-1 (a generous gift from Peter Oettgen) was digested with *Kpn*I and *Xba*I, and the resulting ELF-1 fragment was cloned into pcDNA3. Preparation of ETS-1 and GABP expression vectors was described previously [12], [16].

To generate the Hprt-targeting vector with the rat PF4 promoter (pMP8II-PF4-AcGFP), a 1.1-kb region of the PF4 promoter was amplified by PCR using PF4-luc as a template. The resulting promoter fragment was digested with *Xho*I and *Sac*II, and cloned into the *Xho*I-*Sac*II site of pAcGFP1-1 (Takara, Siga, Japan) to generate PF4-AcGFP. The promoter sequence was verified by automated DNA sequencing. PF4-AcGFP was then digested with *Afl*II and blunt-ended with T4 DNA polymerase. The resulting DNA was digested with *Mlu*I. A 2.1-kb fragment containing PF4 promoter coupled to GFP was purified and cloned between the *Mlu*I and *Pme*I sites of pMP8II [17]. To generate the targeting vector with a mutation of the −51 ETS site (pMP8II-PF4 (−51 ETSmut)-AcGFP), a 170 bp fragments spanning the mutation was PCR amplified using the −51 ETSmut plasmid as a template. The resulting promoter fragment was digested with *Sca*I and *Sac*II, and cloned between the *Sca*I and *Sac*II sites of PF4-AcGFP. The promoter sequence in the obtained plasmid (PF4(−51ETSmut)-AcGFP) was verified by automated DNA sequencing. PF4(−51ETSmut)-AcGFP was then digested with *Afl*II and blunt-ended with T4 DNA polymerase. The resulting DNA was digested with *Mlu*I and 2.1-kb fragment was cloned between the *Mlu*I and *Pme*I sites of pMP8II.

### Cell culture and transient transfection assays

HEL cells were maintained in RPMI 1640 medium supplemented with 10% fetal bovine serum, 100 IU/ml penicillin and 100 µg/ml streptomycin. HepG2 cells were maintained under the same conditions except that Dulbecco's modified Eagle's medium (DMEM) was used. In transient transfection assays with HepG2 cells, 0.5 µg of PF4-luc or hPF4-luc were transfected into 2×10^5^ cells with or without 0.5 µg of each expression vector, using the Lipofectamine 2000 reagent (GIBCO BRL, Gaithersburg, MD). To control for transfection efficiency, the cells were also transfected with 0.5 µg of pβactin-lacZ. In the assay with HEL cells, 7 µg each of the reporter plasmids and pβactin-lacZ were transfected into 1×10^7^ cells by electroporation as described previously [18]. Transfected cells were cultured for 48 hr and assayed for luciferase and β-galactosidase activity. Each assay was performed in duplicate more than three times.

### Electrophoretic mobility shift assay (EMSA)

Nuclear extracts were prepared from HEL cells using Nuclear Extract Kit (Active Motif, Carlsbad, CA). In vitro translated ETS-1, PU.1, FLI-1, ELF-1, and GABP were prepared using the TNT Quick Coupled transcription/translation System (Promega, Madison, WI) and 1 µg of expression vectors. To generate EMSA probes, oligonucleotides were annealed, labeled with T4 polynucleotide kinase and [γ-P^32^] ATP, and purified with G-50 micro columns (Amersham Pharmacia Biotech, Uppsala, Sweden). Binding reactions were carried out using 50 fmol of each probe, 3 to 5 µg nuclear extract or 2 to 4 µl of in vitro translated proteins in binding buffer (2 mM HEPES-NaOH (pH 7.9), 2% glycerol, 0.01 mM EDTA, 0.25 mM DTT, 5 mM KCl, 0.1 mg/ml bovine serum albumin, 5 ng/µl poly dI-dC) for 40 min at 4°C. For competition and supershift assays, competitor oligonucleotides or antibodies were pre-incubated with nuclear extract or in vitro translated proteins for 10 min prior to addition of the probe. Antibodies to ETS-1 (C-20), PU.1 (T-21), FLI-1 (C-19), ELF-1 (C-20), GABPα (H-180), GABPβ1/2 (H-265), and Stat5 (C-17) used as a control antibody were purchased from Santa Cruz Biotechnology (Santa Cruz, CA). Gel electrophoresis was carried out using 4% native polyacrylamide gel and 0.5× TBE buffer at 120 V. All the oligonucleotides sequences are shown in **Table S1**.

### Chromatin immunoprecipitation (ChIP) assay

ChIP assays were performed using a ChIP-IT Express kit (Active Motif, Carlsbad, CA). Briefly, crosslinked genomic DNA was prepared from HEL cells and sheared by sonication using the Digital sonifer model 250 (Branson, Danbury, CT). Resulting DNA-protein complexes were immunoprecipitated using 3 µg of antibodies against FLI-1, ELF-1, GABPα, or control IgG. The precipitated DNA fragments were analyzed by real-time PCR using primers (sequences are shown in **Table S1**) to amplify the promoter region including the −51 ETS sites or GAPDH locus as a control.

### siRNA -mediated knockdown of FLI-1, ELF-1, and GABPα

HEL cells (1×10^7^ cells) were electroporated with 150 to 300 pmol of siRNAs for FLI-1, ELF-1, GABPα or control (sequences are shown in **Table S1**) as described previously [19]. Cells were incubated for 2 days and then processed for total RNA isolation using RNeasy plus mini kit (Qiagen, Germantown, MD). Each cDNA was synthesized from 0.5 µg of total RNA using SuperscriptIII first-strand synthesis system (Invitrogen) and used for real-time RT-PCR analyses. Expression levels of *PF4*, *FLI-1*, *ELF-1*, and *GABPα* were normalized by a *GAPDH* expression level. siRNA target and primer sequences are shown in **Table S1**.

### RT-PCR using cDNA from human megakaryocytes or cell lines

Buffy coat peripheral blood (PB) cells were obtained from volunteer blood donors. PB was collected after written consent that was obtained from the volunteer blood donors. All the protocols were approved by the ethic committees in Japanese Red Cross Osaka Blood Center and Osaka University. AC133^+^ cells were isolated from PB mononuclear cells by a MACS AC133 Cell Isolation Kit (Miltenyi Biotech, Auburn, CA), as described previously [20]. Purified AC133^+^ cells were cultured in IMDM containing 20% human serum, 50 IU/ml penicillin, 50 µg/ml streptomycin, and 10 ng/ml thrombopoietin (TPO) (PeproTech Inc., Rocky Hill, NJ). The same amount of TPO was added to the medium every two days, and half of the medium was replaced with new medium after 6 days of incubation. Cells were incubated for 0, 4, 6, or 10 days and then processed for total RNA using ISOGEN (Nippon gene, Tokyo, Japan). Each cDNA was synthesized from 0.5 to 1 µg of total RNA using ReveTra Ace (Toyobo, Osaka, Japan) and used for real-time RT-PCR analyses. The same procedure was used for the preparation of cDNA from HepG2 and HEL cells. All the primer sequences are shown in **Table S1**.

### Generation and differentiation of HPRT-targeted ES cells

Undifferentiated Hprt-deficient BK4 ES cells (a generous gift from Sarah Bronson) were maintained and propagated on mouse embryonic fibroblast cells in Knockout DMEM (Invitrogen) supplemented with 15% FBS, 1% Glutamax-I (Invitrogen), 100 IU/ml penicillin, 100 µg/ml streptomycin, 0.1 mM MEM non-essential amino acids (Invitrogen), 0.1 mM 2-mercaptoethanol, and 1000 U/ml ESGRO (LIF) (Millipore, Billerica, MA). To generate homologous recombinants, ES cells were electroporated with 25 µg of *Sal*I-linearized targeting vectors (pMP8II-PF4-AcGFP and pMP8II-PF4(−51 ETSmut)-AcGFP). Homologous recombinants were selected in the medium containing HAT (SIGMA, St. Louis, MO) for approximately 10 days, at which time individual colonies were picked for expansion and verification of the desired recombination. At least 2 independent ES cell lines were established for each construct. Established ES cell lines were differentiated into megakaryocytes on OP9 stroma cells by culturing for 8 to 10 days in the medium containing 10 ng/ml TPO according to the method as described previously [21].

### Flow cytometry

Two ES cell lines containing the PF4 promoter, with or without a mutation in the −51 ETS site, and a non-recombined control ES cell line were differentiated into megakaryocytic cells for 8 days. The resulting cells were treated with 0.25% Trypsine and collected. The cells (4×10^5^ cells) were then incubated with phycoerythrin-conjugated rat anti-mouse CD41 antibody (BD pharmingen, Franklin Lakes, NJ) or phycoerythrin-conjugated control rat IgG (BD pharmingen) in phosphate-buffered saline containing 0.5% BSA at 4°C for 30 min. The cells were washed 3 times and analyzed by a flow cytometer.

### DNA sequences for the PF4 promoter

The DNA sequences of the rat, mouse, and human PF4 promoters are obtained from Genbank. Accession numbers are M83070.1, NW_001030791.1, and NT_022778.16 for the rat, mouse, and human promoters, respectively.

### Statistical analyses

The statistical significance of differences of the means was determined by Student *t* test.

## Results

### PF4 promoter activity is mediated by the ETS transcription factors ETS-1, PU.1, and FLI-1

**Figure 1:**
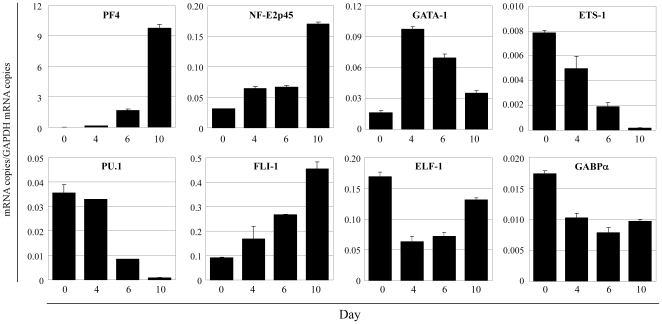
Expression of PF4 and transcription factors during megakaryocytic differentiation. The hematopoietic progenitor (AC133^+^) cells from human peripheral blood were cultured with TPO and differentiated into megakaryocytes. After culturing the cells for 0, 4, 6, and 10 days, the expression of *PF4*, the transcription factors, and *GAPDH* (as an internal control) were analyzed by real-time RT-PCR. Data are represented as the mean ± SE.

We have previously shown that ETS-1 mediates expression of *PF4* [12]. Other ETS factors, including PU.1 and FLI-1, have been implicated in megakaryocyte-specific gene expression [3], [4]. To determine the temporal expression of these genes, we employed a human megakaryocyte differentiation assay. Hematopoietic progenitor (AC133^+^) cells from human peripheral blood cells were differentiated into megakaryocytes by adding thrombopoietin (TPO). The cells were processed 0, 4, 6, and 10 days later for RNA isolation and assayed by real-time PCR for expression of *PF4* and the relevant transcription factors (**Figure 1**). *PF4* demonstrated a time-dependent increase in expression, indicating that AC133^+^ cells were differentiating into the megakaryocytic lineage. A similar pattern was observed for *NF-E2p45*, a transcription factor that has been shown to play an important role during the late stages of megakaryocytic differentiation (reviewed in [22]). The expressions of *ETS-1*, *PU.1*, and *FLI-1* preceded that of *PF4*. Detectable expression of the megakaryocytic transcription factor, GATA-1, coincided with that of PF4. These results are consistent with a role for FLI-1 and PU.1 in regulating *PF4* gene expression.

**Figure 2:**
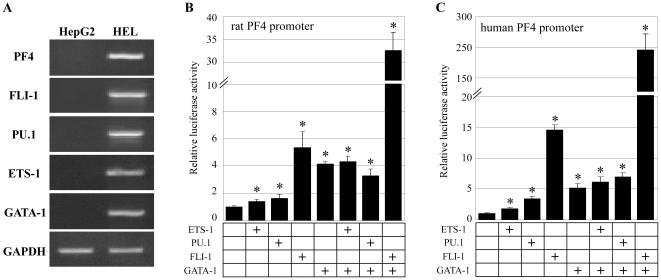
FLI-1 and GATA-1 synergistically activate the PF4 promoter. (**A**) Expression of ETS family proteins and GATA-1 in megakaryocytic HEL cells and non-megakaryocytic HepG2 cells. (**B**, **C**) Coexpression assays were performed in HepG2 cells with expression vectors for ETS-1, PU.1, FLI-1, and GATA-1 and with luciferase constructs including the rat (B) or human (C) PF4 promoter. All the coexpression assays were performed 3 times. Data are represented as the mean ± SE. * p<0.05 between Control and each expression vector.

To determine whether PU.1 or FLI-1 mediate PF4 promoter activity, we employed co-expression assays using HepG2 cells. We chose these cells because it lacks detectable expression of *ETS-1*, *PU.1*, *FLI-1*, and *GATA-1* (**Figure 2A**). Overexpression of ETS-1 or PU.1 resulted in a small induction of the rat PF4 promoter activity, while FLI-1 strongly activated the PF4 promoter (**Figure 2B**). Overexpression of GATA-1 with FLI-1 but not with ETS-1 or PU.1 resulted in synergistic activation of the PF4 promoter (**Figure 2B** **; Figure S1**). A similar pattern was observed with the human PF4 promoter (**Figure 2C**). These results suggest a positive role for FLI-1 in regulating *PF4* gene expression.

### FLI-1 activates the PF4 promoter activity through the −51 ETS site

**Figure 3:**
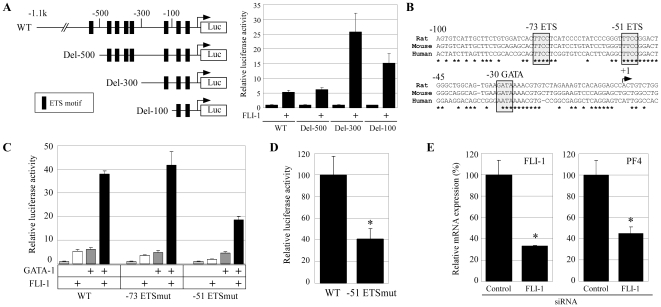
FLI-1 activates the PF4 promoter through the −51 ETS site. (**A**) Schematic representation of the rat PF4 promoter-reporter constructs including the 1.1-kb promoter (wild type) and the deleted promoters (Del-500, Del-300, and Del-100). ETS consensus motifs, including the GGAA core sequence are indicated by closed boxes. The coexpression assay was performed by transfecting these constructs and a FLI-1 expression vector into HepG2 cells. (**B**) Two conserved ETS motifs and a functional GATA motif (reported in [12]) in the 100-bp 5′-upstream promoter. The asterisk indicates the base conserved between 3 species. (**C**) The coexpression assay performed in the HepG2 cells using expression vectors for FLI-1 and GATA-1, and PF4-luc with or without a mutation at the −73 or −51 ETS site. (**D**) The transient transfection assay performed using PF4-luc with or without a mutation at the −51 ETS site and the megakaryocytic HEL cells. * p<0.05 between the wild type and the mutant promoter. (**E**) Control or FLI-1 siRNA was transfected into HEL cells. The expression levels of PF4 and FLI-1 mRNA in the transfected cells were measured by real-time RT-PCR. All the coexpression and siRNA assays were performed 3 times. Data are represented as the mean ± SE. * p<0.05 between Control and FLI-1 siRNA.

In order to identify the FLI-1 binding site that regulates PF4 promoter activity, we cotransfected HepG2 cells with progressively shortened 5′-deletion fragments of the PF4 promoter. As shown in **Figure 3A**, deletion constructs containing 500, 300, and 100 bp of the upstream promoter region (Del-500, Del-300, and Del-100, respectively) were all activated by FLI-1. These findings suggest that the FLI-1-responsive DNA element is contained within 100 bp of the transcriptional start site. A comparative analysis of this region revealed 2 highly conserved ETS consensus elements at −73 and −51 (**Figure 3B**). To determine whether 1 or both sites were responsible for mediating the positive effect of FLI-1 on PF4 promoter activity, we co-transfected HepG2 cells with a FLI-1 expression vector and a wild type or a mutant PF4 promoter-reporter plasmid containing a mutation in the −73 or −51 ETS sites (−73 ETSmut and −51 ETSmut, respectively). Mutation of the −51 ETS site resulted in a significant reduction in FLI-1-mediated promoter activation, while mutation of the −73 ETS had little effect (**Figure 3C**). Consistent with this result, the mutation of the −51 ETS site also resulted in a significant reduction in synergistic promoter activation mediated by FLI-1 and GATA-1, while the mutation of the −73 ETS had no effect (**Figure 3C**). A similar result was obtained with an assay using Del-100, with or without a mutation in the −73 or −51 ETS site (**Figure S2**). These results indicated that the −51 ETS site is a FLI-1 responsive element and important for synergistic promoter activation by FLI-1 and GATA-1. A mutation of the −51 ETS site resulted in a significant decrease of the promoter activity in the *PF4*-expressing megakaryocytic HEL cell (**Figure 3D**). To determine whether FLI-1 plays a role in mediating the expression of the endogenous *PF4* gene, siRNA against FLI-1 was transfected into HEL cells. FLI-1 siRNA resulted in 67% and 56% reduction of FLI-1 and PF4 mRNA expression, respectively (**Figure 3E**). Together, these data suggest that FLI-1 regulates *PF4* gene expression through the −51 ETS site.

### ETS transcription factors bind to the −51 ETS site

**Figure 4:**
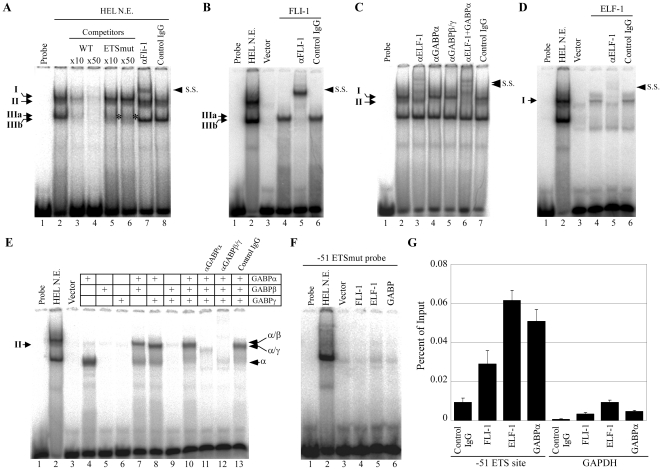
FLI-1, ELF-1, and GABP bind to the −51 ETS site. (**A**) EMSA performed with the −51 ETS probe. Nuclear extracts from HEL cells were added to lanes 2–8. Competition assays were performed with wild type (lanes 3 and 4) or −51 ETSmut (lanes 5 and 6) competitors. The supershift assays were performed with the antibody against FLI-1 and control antibody (lanes 7 and 8). Four shifted bands were labeled I, II, IIIa and IIIb. Asterisk indicates the specific shifted band IIIa. (**B**) EMSA was performed with the in vitro translated FLI-1 and control protein (Vector) (lanes 3 and 6). The supershift assay was performed with the antibody against FLI-1 and control antibody (lanes 5 and 6). (**C**) The supershift assay was performed with antibodies against ELF-1 and GABP or control antibody (lanes 3–7). GABPβ/γ antibody recognizes both GABPβ and its splicing isoform GABPγ. (**D**) EMSA was performed with the in vitro translated ELF-1 and control protein (lanes 3–6). The supershift assay was performed with the antibody against ELF-1 and control antibody (lanes 5 and 6). (**E**) EMSA was performed with the in vitro translated GABP (GABP α, β, and γ) and control protein (lanes 3–13). The supershift assay was performed with the antibody against GABP and control antibody. Shifted bands derived from GABPα, GABPα/β and GABPβ/γ complexes are labeled α, α/β, and α/γ, respectively. (**F**) EMSA was performed with the −51 ETSmut probe and in vitro translated FLI-1, ELF-1, GABP, and control protein. (**G**) ChIP assay was performed with HEL cells by using antibodies against ETS factors or control antibody. Immunoprecipitated DNA was measured by real-time PCR with primers to amplify the human PF4 promoter region, including the −51 ETS site, or the GAPDH locus as a negative control region. A representative of 3 independent experiments is shown.

To determine which ETS factors bind to the −51 ETS site in megakaryocytic cells, electrophoretic mobility shift assay (EMSA) was performed using a probe spanning the −51 ETS site and nuclear extract from HEL cells. Four shifted bands were observed (**Figure 4A**; Band I, II, IIIa, and IIIb, respectively). Band IIIa and IIIb were detected almost at the same position. Band I, II, and IIIa were inhibited by the addition of a cold wild type competitor probe, but not a mutant competitor probe (**Figure 4A**; lanes 3–6). Band IIIb was inhibited by the addition of both wild type and mutant competitor probes, suggesting that band IIIb is not derived from a protein which specifically binds to the −51 ETS site. Incubation of the reaction mixture with anti-FLI-1 antibody, but not control antibody resulted in a supershifted band (**Figure 4A**; lanes 7 and 8). Consistent with these results, EMSA using in vitro translated FLI-1 indicated that FLI-1 binds to the −51 ETS site and that a FLI-1-DNA complex is observed to have a size similar to that of band IIIa (**Figure 4B**).

The above results suggested that ETS factors other than FLI-1 were involved in the DNA-protein complexes corresponding to band I and II. To identify these factors, we performed supershift assays with antibodies against other ETS proteins. Band I was inhibited by addition of anti-ELF-1 antibody, while band II was inhibited by anti-GABP antibody (**Figure 4C**; lanes 3–5). Control IgG had no effect on the DNA-protein complexes (**Figure 4C**; lane 7). EMSA was performed with in vitro translated ELF-1 and GABP. GABP binds as a complex consisting of heterodimers of GABPα and GABPβ or γ (reviewed in [23]). The recombinant ELF-1 resulted in a DNA-protein complex that migrated at the same position as band I (**Figure 4D**; lane 4). Anti-ELF-1 antibody inhibited the complex and resulted in a weak super-shift band (**Figure 4D**; lane 5). The recombinant GABPα together with GABPβ and/or GABPγ resulted in DNA-protein complexes that corresponded in size to band II (**Figure 4E**). FLI-1, ELF-1, and GABP (containing recombinant GABPα, GABPβ and GABPγ) did not bind to a probe containing a mutation of the −51 ETS site, and this multiple protein binding of 3 ETS family proteins was not observed with the −73 ETS site (**Figure 4F** **; Figure S3**). Consistent with the EMSA, a chromatin immunoprecipitation (ChIP) assay using HEL cells also demonstrated the bindings of FLI-1, ELF-1, and GABP to the −51 ETS site (**Figure 4G**). Together, these findings suggest that FLI-1, ELF-1, and GABP bind to the −51 ETS site in the PF4 promoter.

### ELF-1 and GABP regulate the *PF4* gene expression through the −51 ETS site

**Figure 5:**
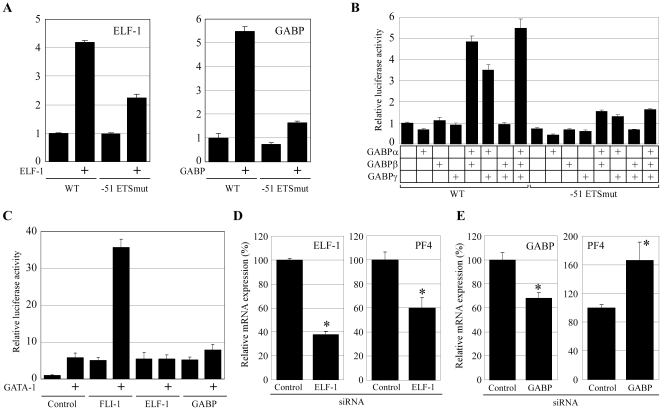
FLI-1, ELF-1, and GABP activate the PF4 promoter through the −51 ETS site. (**A**) Coexpression assays were performed in HepG2 cells by using the expression vectors for FLI-1, ELF-1, and GABP (consisting of α, β and γ), and luciferase constructs containing the 1.1-kb promoter with or without a mutation in the −51ETS site. (**B**) Coexpression assay was performed with expression vectors for GABPα, β, and γ, and luciferase constructs containing 1.1-kb promoter with or without a mutation in the −51 ETS site. (**C**) Coexpression assay was performed with GATA-1 expression vector and expression vectors for FLI-1, ELF-1 and GABP. In all experiments, an empty vector (pcDNA3) was used as a control vector. All of the coexpression assays were performed 3 times. Data are represented as the mean ± SE. (**D**, **E**) Control siRNA, or siRNA for ELF-1 or GABPα was transfected into HEL cells. The expression levels of PF4, and ELF-1 or GABPα mRNAs in the transfected cells were measured by real-time PCR. * p<0.05 between Control and ELF-1 or GABP siRNA.

To determine the role of ELF-1 and GABP in mediating PF4 promoter activity, coexpression assays were performed. Overexpression of ELF-1 or GABP (GABPα, β, and γ) resulted in strong activation of the wild-type promoter, but not the −51 ETS mutant promoter in HepG2 cells (**Figure 5A**). The effect of GABP on promoter activation was recapitulated by combined expression of either GABPα/β or GABPα/γ (**Figure 5B**). To determine whether ELF-1 and/or GABP functionally interact with GATA-1 to mediate promoter activity, co-transfection assays in HepG2 cells were performed. Unlike FLI-1, ELF-1 and GABP did not synergistically activate the PF4 promoter with GATA-1 (**Figure 5C**). These results demonstrated that ELF-1 and GABP activate the PF4 promoter through the −51 ETS site while these factors cannot synergistically activate the promoter with GATA-1.

To investigate whether ELF-1 and/or GABP regulate the endogenous *PF4* gene expression in megakaryocytic cells, siRNA against ELF-1 or GABP were transfected into HEL cells. ELF-1 siRNA resulted in 62% and 40% reduction in ELF-1 and PF4 mRNA expression (**Figure 5D**). On the other hand, GABP siRNA resulted in 32% reduction of *GABP* and 66% increase of PF4 mRNA expression (**Figure 5E**). These results indicated that both ELF-1 and GABP regulates the *PF4* gene expression. Although the result of co-expression assay showed that both ELF-1 and GABP are positive regulators for the *PF4* gene expression, siRNA knockdown of GABP led to increased expression of *PF4* in HEL cells. We speculated that this controversial effect was observed because the decrease of GABP promoted FLI-1 binding to the −51 ETS sites. In fact, EMSA using recombinant proteins showed competitive binding of FLI-1, ELF-1, or GABP at the −51 ETS site (**Figure S4**). Thus, these data suggest that FLI-1, ELF-1, and GABP competitively bind to the −51 ETS sites and regulate *PF4* gene expression.

### Expression patterns of FLI-1, ELF-1, and GABP in human megakaryocytic differentiation

To investigate and compare the temporal expression patterns of the ETS factors FLI-1, ELF-1, and GABP during megakaryocytic differentiation, mRNA levels of ELF-1 and GABPα were measured by real-time PCR (using the same cDNA prepared in Figure 1). As shown in **Figure 1**, both ELF-1 and GABPα were expressed during megakaryocytic differentiation. However, unlike the expression pattern of FLI-1 expressions of *ELF-1* and *GABPα* had decreased by day 4. This finding suggests that while all 3 ETS factors may play a role in mediating *PF4* expression, they likely function in temporally distinct ways.

### The −51 ETS site is important for PF4 promoter activation in differentiating megakaryocytes

**Figure 6:**
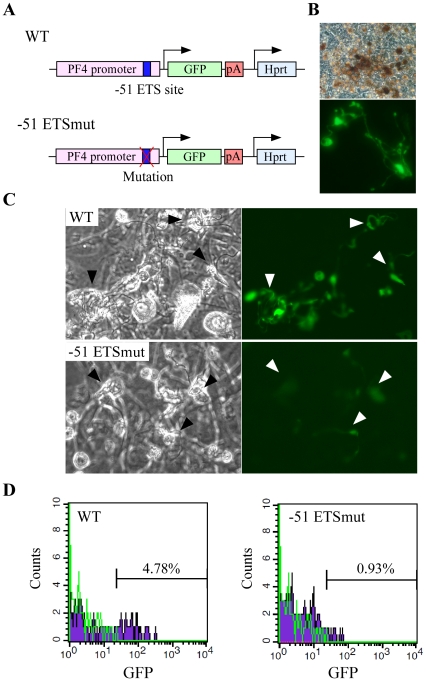
The −51 ETS site is important for PF4 promoter activity in differentiating megakaryocytes. (**A**) Two ES cell lines with transgenes containing PF4 promoter with or without a mutation in the −51 ETS site were prepared. The genomic DNA sequence between these ES cell lines differed only at the −1 ETS site. (**B**) Differentiated cells from ES cells with a wild type promoter were stained for acetylcholine esterase (upper panel). These cells included GFP positive proplatelets (lower panel). (**C**) GFP expression level was compared between 2 cell lines with WT and −51 ETSmut promoters. The arrowheads indicate proplatelets. (**D**) The ratio of GFP expressing CD41-positive cells derived from ES cell lines containing the wild type or −51 ETSmut promoter was analyzed by a flow cytometer.

To confirm a role for the −51 ETS site in mediating the *PF4* gene expression in megakaryocytes, the wild type or the −51 ETSmut PF4 promoter was coupled to GFP. A single copy of the resulting transgenic cassette was targeted to the Hprt locus in ES cells by homologous recombination (**Figure S5A**; **Figure 6A**). Two correctly targeted recombinant clones for each construct were differentiated into megakaryocytic lineage by culturing with OP9 stroma cells and TPO. At day 10, a number of differentiated cells were positive for acetylcholine esterase activity (**Figure 6B** upper panel), and 30% of the cells were positive for CD41 (data not shown). In addition, proplatelets were also observed (**Figure 6B** lower panel), suggesting differentiation into the megakaryocytic lineage. Megakaryocytes and proplatelets from cells carrying the wild type PF4 promoter had strong GFP expression, while those from −51 ETSmut showed significantly decreased GFP expression (**Figure 6C** **; Figure S5B**). FACS analysis demonstrated that the mutation of the −51 ETS site significantly decreased the number of GFP positive megakaryocytic cells (**Figure 6D**). These observations suggest that the −51 ETS site is important for the activation of the PF4 promoter during megakaryocytic differentiation.

## Discussion

Multiple ETS binding sites are often found in the megakaryocyte-specific gene promoters. However, it has not been clearly shown how multiple ETS binding sites regulate megakaryocyte-specific genes. The rat PF4 promoter contains more than 20 ETS core sequences in it. In this study, we identified a novel, functional ETS binding site (−51 ETS site) in the PF4 promoter. To evaluate the physiological function of the −51 ETS site for the PF4 promoter activity in megakaryocytes, we developed and used a novel reporter gene assay using homologously recombined mouse ES cells (**Figure 6** **; Figure S5**). It has been difficult to analyze the promoter activity in intact megakaryocytes because of the small number of megakaryocytes in vivo and the technical difficulty of transient transfection of plasmids into megakaryocytes and proplatelets. Our system enables the promoter activity in normal differentiating megakaryocytes and proplatelets to be evaluated. Using this system we evaluated the function of the −51 ETS site using 2 independent ES cell lines containing a wild type or mutant PF4 promoter coupled to the GFP gene. The insertion of a singly copy transgene into the Hprt locus allowed to compare the expression levels of the reporter gene between ES cell lines in the same genomic DNA context [24]. By differentiating these ES cell lines into megakaryocytic lineage, we succeeded in demonstrating that the −51 ETS site is important for the activation of *PF4* gene expression in megakaryocytes and proplatelets. This reporter assay system should be a powerful tool for analyzing physiological promoter function in normal megakaryocytes and proplatelets.

Our data demonstrate that multiple ETS family proteins regulate the *PF4* gene expression. EMSA and coexpression assays indicate that FLI-1, ELF-1, and GABP bind to the −51 ETS site and activate the PF4 promoter. In addition, the ChIP assay showed that FLI-1, ELF-1, and GABPα bind to the −51 ETS site in vivo. ELF-1 was originally described as a regulator of T-cell specific genes [25]. To our knowledge, this is the first study to demonstrate a role for ELF-1 in mediating the megakaryocyte-specific gene expression. GABP consists of 2 subunits, GABPα and GABPβ (or its splicing isoform, GABPγ). GABPα contains the ETS DNA-binding domain, while GABPβ is required for nuclear translocation and transactivation. Mammalian GABP is ubiquitously expressed in all tissues and has been implicated in several critical cellular processes such as cellular differentiation, cell cycle, cell survival and mitochondrial respiration [23]. In addition to regulating the expression of housekeeping genes, GABP has been shown to regulate the expression of cell-type specific genes in several distinct lineages, including myeloid cells, lymphocytes, mast cells and endothelial cells [16], [23]. A few reports indicate the importance of GABP in the regulation of megakaryocyte-specific genes (e.g., *GPIIb* and *cMpl*) [26], [27]. Comparing with FLI-1, ELF-1, and GABP, ETS-1 has a small effect on the PF4 gene expression in the co-expression assay using HepG2 cells, although we have previously demonstrated that ETS-1 activates the PF4 promoter [12]. This difference may be caused by the difference in the cell types used in the co-expression assay. ETS-1 is known to inhibit its DNA binding via the autoinhibitory domain, and its DNA binding is enhanced by interacting with other transcription factors, such as CBFβ [28]. We speculated that some interacting protein that is expressed in HEL cells but not HepG2 cells is needed for the strong promoter activation by ETS-1.

It has been reported that multiple transcription factors can bind to the same site and regulate gene expression. Recent genome-wide ChIP-seq analyses have also shown an overlap in the chromatin regions commonly bound by different members of the ETS family [29], [30], [31]. In some cases, a transcriptional activator competes with a suppressor for binding to the same site. This mechanism enables positive or negative regulation of promoter activity through the same site. However, in our reporter gene assays, individual overexpression of FLI-1, ELF-1, and GABP resulted in similar levels of PF4 promoter activity. This suggests that all these factors are transcriptional activators of the PF4 promoter. The question is: why is the PF4 promoter regulated by multiple transcriptional activators through the same site? In the coexpression assay, FLI-1 but not ELF-1 and GABP synergistically activated the PF4 promoter with GATA-1 (**Figure 5C**). One potential mechanism to explain this synergistic activation may be a direct interaction between FLI-1 and GATA-1 on the GATA-1 binding site, because FLI-1 expression enhanced promoter activation by GATA-1 without binding to the −51 ETS site (**Figure 3C** **; Figure S2**). Alternatively, GATA-1 may enhance the FLI-1 binding to the other ETS site, such as the −71 ETS site. In either case, this synergistic activation by FLI-1 and GATA-1 suggests distinct roles for the ETS family proteins in mediating gene expression by interacting with other transcription factors. The siRNA knockdown of GABPα slightly increased the *PF4* gene expression although the overexpression of GABP itself activated the promoter activity in the co-expression assay (**Figure 5A, E**). This controversial observation also suggests the possibility that FLI-1, ELF-1, and GABP competitively bind to the −51 ETS site, and that the relative expression levels of these 3 factors are important for the regulation of the *PF4* gene expression. Our real-time PCR analysis indicated that the expression of FLI-1 increased during megakaryocytic differentiation, whereas the expression of ELF-1 and GABP decreased at day 4 and did not drastically change at days 6 and day 10 (**Figure 1**). This suggests the possibility that FLI-1 plays a role in the late stage of megakaryocytic differentiation, whereas ELF-1 and GABP are necessary during the early stage. Consistent with this hypothesis, the importance of the FLI-1 at the late stage of megakaryocytic differentiation and gene expression was demonstrated by inducible FLI-1 knock out and FLI-1 mutant mice [32], [33]. Furthermore, Pang et al. reported that GABP regulates megakaryocyte-specific genes that are expressed at the early differentiation stage, while FLI-1 regulates genes expressed at the late stage [27]. In the regulation of the late megakaryocytic marker *PF4* either GABP or ELF-1 may bind to the −51 ETS site and moderately activate the promoter at the early stage, whereas FLI-1 may displace them and strongly activate the promoter with GATA-1 at the late stage. Our EMSA data indicate the possibility that FLI-1 displaces ELF-1 and GABP from the −51 ETS site (**Figure S4**). Together, these results implicate the differentiation stage-specific regulation of *PF4* gene expression by multiple ETS factors.

Inconsistent with our result, Pang's group demonstrated that GABP synergistically activates the GPIIb promoter with GATA-1 under the existence of FOG-1. This suggests the possibilities that FOG-1 is essential for synergistic activation of the PF4 promoter by GABP and GATA-1 and that synergistic activation by GABP and GATA-1 does not occur on the PF4 promoter. More detailed analyses about ETS family proteins and their functionally associated proteins will be important to understand megakaryocyte-specific and differentiation stage-specific PF4 gene expression.

## Supporting Information

Figure S1 **Expression of transcription factors in transient transfection assay.** Expression levels of the transcription factors and GAPDH (as a control) in the transfected HepG2 cells were analyzed by western blotting. The asterisks indicate bands derived from FLI-1 between non-specific bands.
(TIF)

Figure S2 **Activation of the PF4 promoter by FLI-1 through the −51 ETS site.** Coexpression assay was performed in HepG2 cells by using FLI-1 and GATA-1 expression vectors and PF4-luc with or without a mutation in the −73 or −51 ETS site.
(TIF)

Figure S3 **Binding of FLI-1, ELF-1, or GABP to the −73 ETS site.** EMSA was performed with the −73 ETS or −51 ETS probe, and FLI-1, ELF-1, and GABP prepared by in vitro translation. The arrows indicate the shifted bands derived from FLI-1 (F), ELF-1 (E) and GABP (G). The DNA sequences of oligonucleotides for the −73 ETS probe are shown in Table S1.
(TIF)

Figure S4 **Competitive binding of FLI-1, and ELF-1 or GABP to the −51 ETS site.** (**A**) EMSA was performed with the −51 ETS probe, and in the protein mixture containing ELF-1 and various amounts of FLI-1. The arrows indicate the shifted bands derived from ELF-1 (E) and FLI-1 (F). (**B**) EMSA was performed with the −51 ETS probe, and the protein mixture containing GABPα and various amounts of FLI-1. The arrows indicate the shifted bands derived from GABPα (G) and FLI-1 (F).
(TIF)

Figure S5 **Promoter activity of the wild type or mutant PF4 promoter during megakaryocytic differentiation.** (**A**) The transgene was inserted into the Hprt locus by homologous recombination. (**B**) Two ES cell lines with transgenes containing the PF4 promoter with or without a mutation in the −51 ETS site were differentiated into megakaryocytic lineage. The GFP expression level was compared between the 2 cell lines from days 6 to 12.
(TIF)

Table S1 **Sequences of the oligonucleotides.**
(XLS)

## References

[1] Szalai G, LaRue AC, Watson DK (2006). Molecular mechanisms of megakaryopoiesis.. Cell Mol Life Sci.

[2] Wasylyk B, Hahn SL, Giovane A (1993). The Ets family of transcription factors.. Eur J Biochem.

[3] Eisbacher M, Holmes ML, Newton A, Hogg PJ, Khachigian LM (2003). Protein-protein interaction between Fli-1 and GATA-1 mediates synergistic expression of megakaryocyte-specific genes through cooperative DNA binding.. Mol Cell Biol.

[4] Doubeikovski A, Uzan G, Doubeikovski Z, Prandini MH, Porteu F (1997). Thrombopoietin-induced expression of the glycoprotein IIb gene involves the transcription factor PU.1/Spi-1 in UT7-Mpl cells.. J Biol Chem.

[5] Zhang C, Gadue P, Scott E, Atchison M, Poncz M (1997). Activation of the megakaryocyte-specific gene platelet basic protein (PBP) by the Ets family factor PU.1.. J Biol Chem.

[6] Rekhtman N, Radparvar F, Evans T, Skoultchi AI (1999). Direct interaction of hematopoietic transcription factors PU.1 and GATA-1: functional antagonism in erythroid cells.. Genes Dev.

[7] Zhang P, Behre G, Pan J, Iwama A, Wara-Aswapati N (1999). Negative cross-talk between hematopoietic regulators: GATA proteins repress PU.1.. Proc Natl Acad Sci U S A.

[8] Zhang P, Zhang X, Iwama A, Yu C, Smith KA (2000). PU.1 inhibits GATA-1 function and erythroid differentiation by blocking GATA-1 DNA binding.. Blood.

[9] Stopka T, Amanatullah DF, Papetti M, Skoultchi AI (2005). PU.1 inhibits the erythroid program by binding to GATA-1 on DNA and creating a repressive chromatin structure.. Embo J.

[10] Rekhtman N, Choe KS, Matushansky I, Murray S, Stopka T (2003). PU.1 and pRB interact and cooperate to repress GATA-1 and block erythroid differentiation.. Mol Cell Biol.

[11] Hong W, Kim AY, Ky S, Rakowski C, Seo SB (2002). Inhibition of CBP-mediated protein acetylation by the Ets family oncoprotein PU.1.. Mol Cell Biol.

[12] Minami T, Tachibana K, Imanishi T, Doi T (1998). Both Ets-1 and GATA-1 are essential for positive regulation of platelet factor 4 gene expression.. Eur J Biochem.

[13] Okada Y, Nagai R, Sato T, Matsuura E, Minami T (2003). Homeodomain proteins MEIS1 and PBXs regulate the lineage-specific transcription of the platelet factor 4 gene.. Blood.

[14] Okada Y, Matsuura E, Nagai R, Sato T, Watanabe A (2003). PREP1, MEIS1 homolog protein, regulates PF4 gene expression.. Biochem Biophys Res Commun.

[15] Okada Y, Matsuura E, Tozuka Z, Nagai R, Watanabe A (2004). Upstream stimulatory factors stimulate transcription through E-box motifs in the PF4 gene in megakaryocytes.. Blood.

[16] Okada Y, Yano K, Jin E, Funahashi N, Kitayama M (2007). A three-kilobase fragment of the human Robo4 promoter directs cell type-specific expression in endothelium.. Circ Res.

[17] Evans V, Hatzopoulos A, Aird WC, Rayburn HB, Rosenberg RD (2000). Targeting the Hprt locus in mice reveals differential regulation of Tie2 gene expression in the endothelium.. Physiol Genomics.

[18] Ravid K, Doi T, Beeler DL, Kuter DJ, Rosenberg RD (1991). Transcriptional regulation of the rat platelet factor 4 gene: interaction between an enhancer/silencer domain and the GATA site.. Mol Cell Biol.

[19] Walters DK, Goss VL, Stoffregen EP, Gu TL, Lee K (2006). Phosphoproteomic analysis of AML cell lines identifies leukemic oncogenes.. Leuk Res.

[20] Matsumoto K, Yasui K, Yamashita N, Horie Y, Yamada T (2000). In vitro proliferation potential of AC133 positive cells in peripheral blood.. Stem Cells.

[21] Fujimoto TT, Kohata S, Suzuki H, Miyazaki H, Fujimura K (2003). Production of functional platelets by differentiated embryonic stem (ES) cells in vitro.. Blood.

[22] Shivdasani RA (2001). Molecular and transcriptional regulation of megakaryocyte differentiation.. Stem Cells.

[23] Rosmarin AG, Resendes KK, Yang Z, McMillan JN, Fleming SL (2004). GA-binding protein transcription factor: a review of GABP as an integrator of intracellular signaling and protein-protein interactions.. Blood Cells Mol Dis.

[24] Okada Y, Jin E, Nikolova-Krstevski V, Yano K, Liu J (2008). A GABP-binding element in the Robo4 promoter is necessary for endothelial expression in vivo.. Blood.

[25] Thompson CB, Wang CY, Ho IC, Bohjanen PR, Petryniak B (1992). cis-acting sequences required for inducible interleukin-2 enhancer function bind a novel Ets-related protein, Elf-1.. Mol Cell Biol.

[26] Kamura T, Handa H, Hamasaki N, Kitajima S (1997). Characterization of the human thrombopoietin gene promoter. A possible role of an Ets transcription factor, E4TF1/GABP.. J Biol Chem.

[27] Pang L, Xue HH, Szalai G, Wang X, Wang Y (2006). Maturation stage-specific regulation of megakaryopoiesis by pointed-domain Ets proteins.. Blood.

[28] Gu TL, Goetz TL, Graves BJ, Speck NA (2000). Auto-inhibition and partner proteins, core-binding factor beta (CBFbeta) and Ets-1, modulate DNA binding by CBFalpha2 (AML1).. Mol Cell Biol.

[29] Hollenhorst PC, Chandler KJ, Poulsen RL, Johnson WE, Speck NA (2009). DNA specificity determinants associate with distinct transcription factor functions.. PLoS Genet.

[30] Wei GH, Badis G, Berger MF, Kivioja T, Palin K (2010). Genome-wide analysis of ETS-family DNA-binding in vitro and in vivo.. Embo J.

[31] Wilson NK, Foster SD, Wang X, Knezevic K, Schutte J (2010). Combinatorial transcriptional control in blood stem/progenitor cells: genome-wide analysis of ten major transcriptional regulators.. Cell Stem Cell.

[32] Starck J, Weiss-Gayet M, Gonnet C, Guyot B, Vicat JM (2010). Inducible Fli-1 gene deletion in adult mice modifies several myeloid lineage commitment decisions and accelerates proliferation arrest and terminal erythrocytic differentiation.. Blood.

[33] Moussa O, LaRue AC, Abangan RS, Williams CR, Zhang XK (2010). Thrombocytopenia in mice lacking the carboxy-terminal regulatory domain of the Ets transcription factor Fli1.. Mol Cell Biol.

