# Supplementary figures and images for: Multiple ETS Family Proteins Regulate PF4 Gene Expression by Binding to the Same ETS Binding Site
Source: PLoS One. 2011 Sep 12;6(9):e24837. doi: 10.1371/journal.pone.0024837 (PMC3171469; doi:10.1371/journal.pone.0024837)

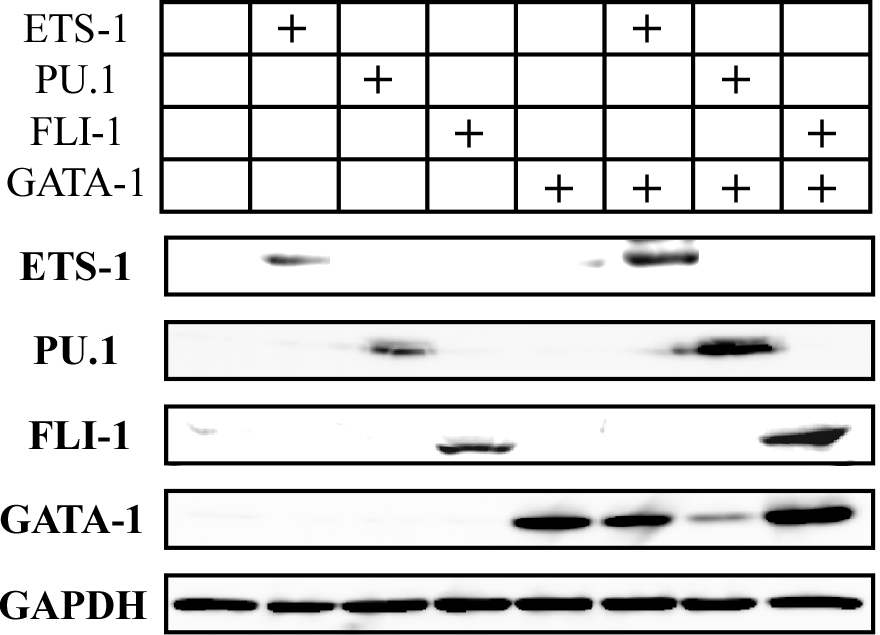

Supplement: Figure S1 — Expression of transcription factors in transient transfection assay. Expression levels of the transcription factors and GAPDH (as a control) in the transfected HepG2 cells were analyzed by western blotting. The asterisks indicate bands derived from FLI-1 between non-specific bands. (TIF) [file pone.0024837.s001.tif]

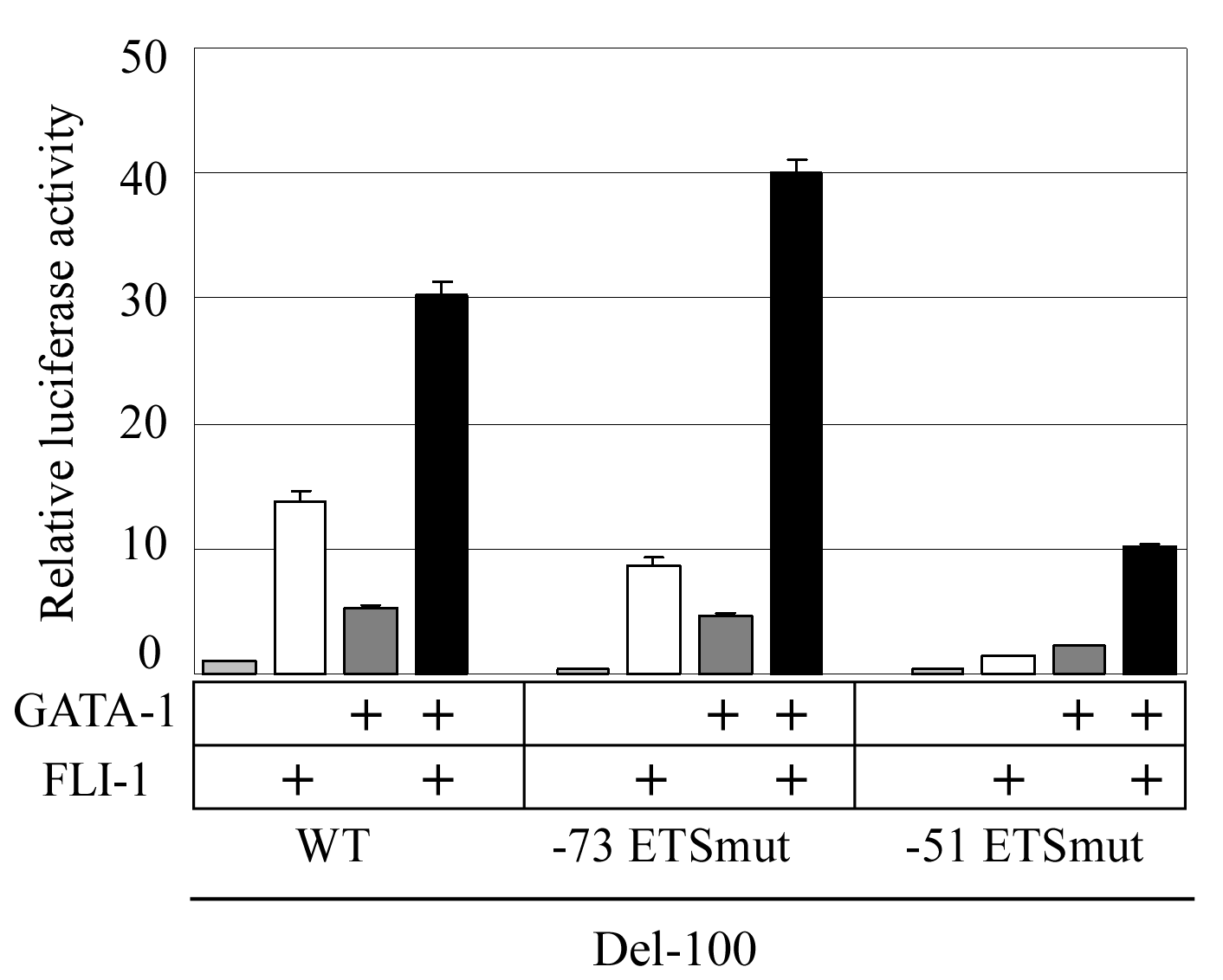

Supplement: Figure S2 — Activation of the PF4 promoter by FLI-1 through the −51 ETS site. Coexpression assay was performed in HepG2 cells by using FLI-1 and GATA-1 expression vectors and PF4-luc with or without a mutation in the −73 or −51 ETS site. (TIF) [file pone.0024837.s002.tif]

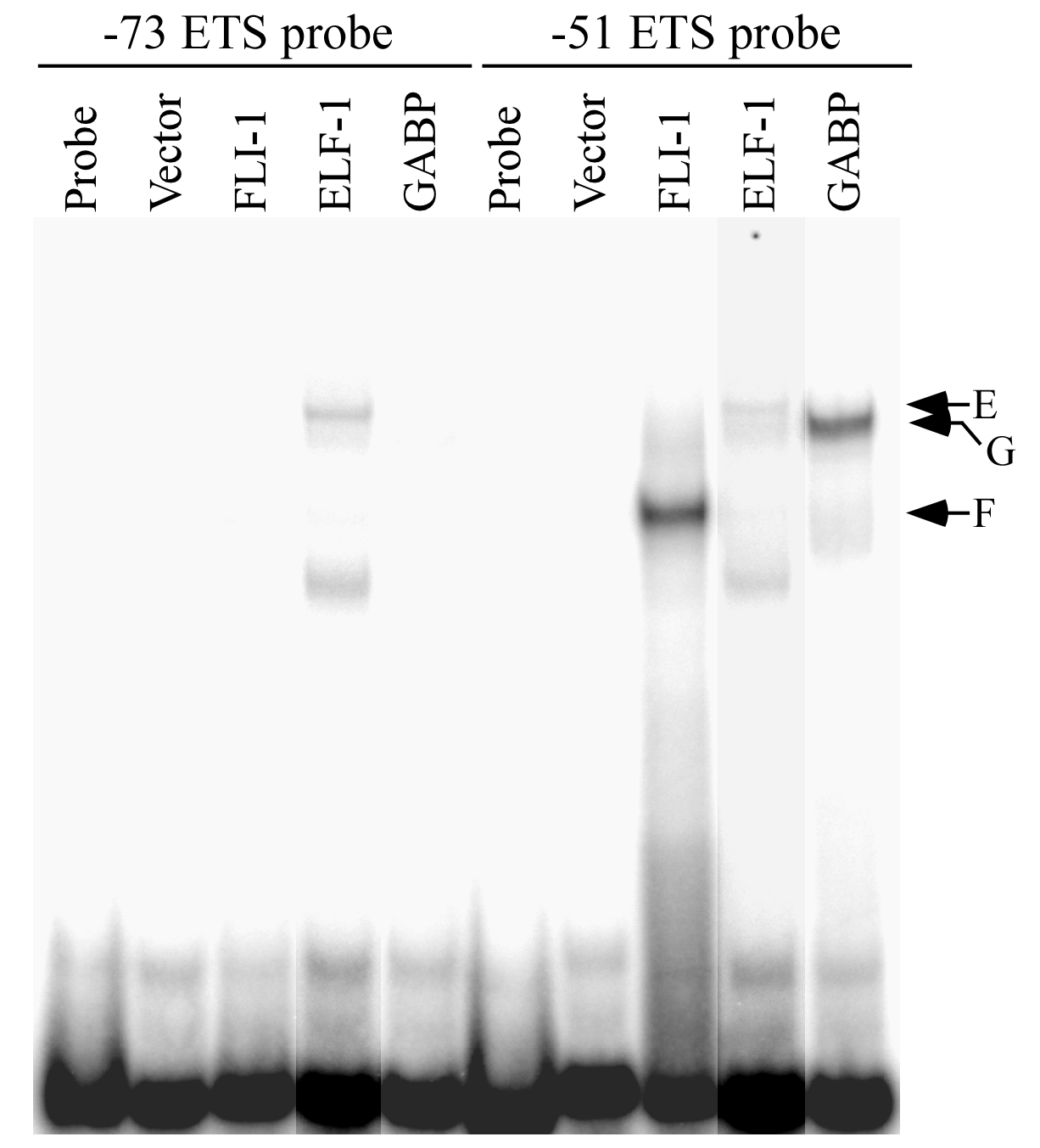

Supplement: Figure S3 — Binding of FLI-1, ELF-1, or GABP to the −73 ETS site. EMSA was performed with the −73 ETS or −51 ETS probe, and FLI-1, ELF-1, and GABP prepared by in vitro translation. The arrows indicate the shifted bands derived from FLI-1 (F), ELF-1 (E) and GABP (G). The DNA sequences of oligonucleotides for the −73 ETS probe are shown in Table S1. (TIF) [file pone.0024837.s003.tif]

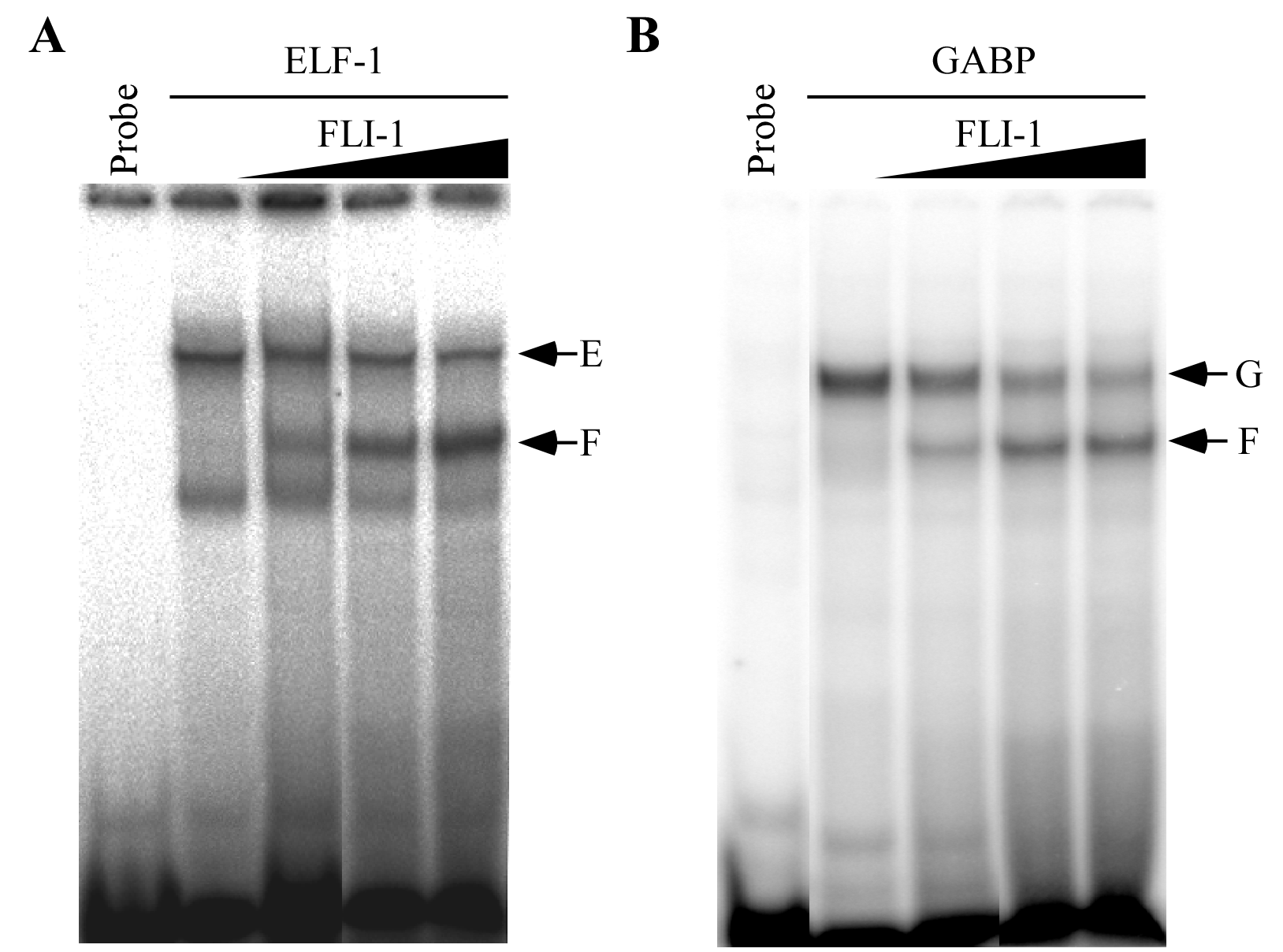

Supplement: Figure S4 — Competitive binding of FLI-1, and ELF-1 or GABP to the −51 ETS site. (A) EMSA was performed with the −51 ETS probe, and in the protein mixture containing ELF-1 and various amounts of FLI-1. The arrows indicate the shifted bands derived from ELF-1 (E) and FLI-1 (F). (B) EMSA was performed with the −51 ETS probe, and the protein mixture containing GABPα and various amounts of FLI-1. The arrows indicate the shifted bands derived from GABPα (G) and FLI-1 (F). (TIF) [file pone.0024837.s004.tif]

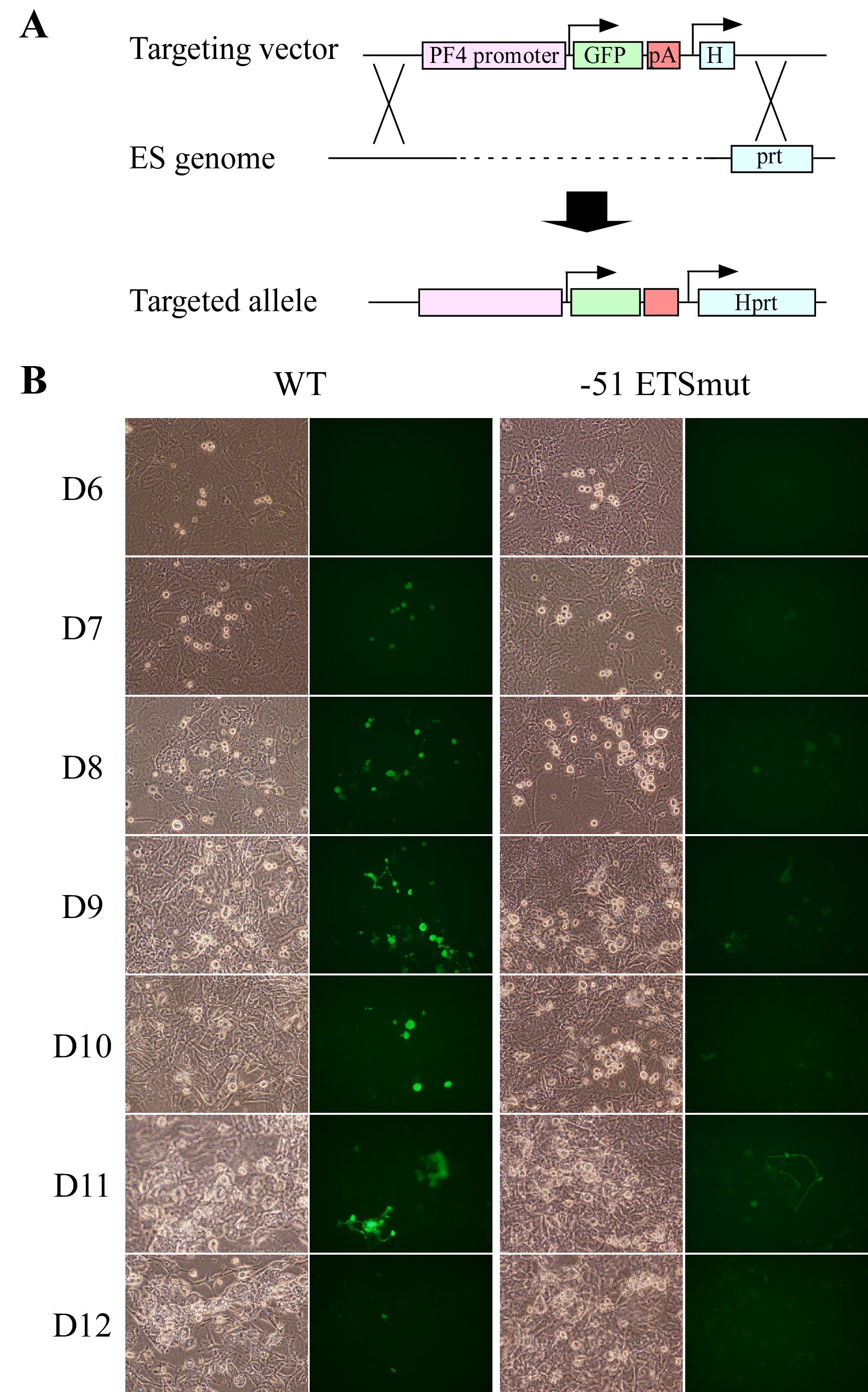

Supplement: Figure S5 — Promoter activity of the wild type or mutant PF4 promoter during megakaryocytic differentiation. (A) The transgene was inserted into the Hprt locus by homologous recombination. (B) Two ES cell lines with transgenes containing the PF4 promoter with or without a mutation in the −51 ETS site were differentiated into megakaryocytic lineage. The GFP expression level was compared between the 2 cell lines from days 6 to 12. (TIF) [file pone.0024837.s005.tif]
